# Succinate dehydrogenase inversely regulates red cell distribution width and healthy life span in chronically hypoxic mice

**DOI:** 10.1172/jci.insight.158737

**Published:** 2022-09-08

**Authors:** Bora E. Baysal, Abdulrahman A. Alahmari, Tori C. Rodrick, Debra Tabaczynski, Leslie Curtin, Mukund Seshadri, Drew R. Jones, Sandra Sexton

**Affiliations:** 1 Department of Pathology and; 2 Department of Pharmacology and Therapeutics, Roswell Park Comprehensive Cancer Center, Buffalo, New York, USA.; 3 Department of Medical Laboratory Sciences, Prince Sattam Bin Abdulaziz University, Alkharj, Saudi Arabia.; 4 Metabolomics Laboratory, NYU Langone Health, New York, New York, USA.; 5 Department of Molecular and Cellular Biology,; 6 Laboratory Animal Shared Resource, and; 7 Dentistry and Maxillofacial Prosthetics service and Department of Oral Oncology, Roswell Park Comprehensive Cancer Center, Buffalo, New York, USA.

**Keywords:** Hematology, Pulmonology, Hypoxia, Mitochondria

## Abstract

Increased red cell distribution width (RDW), which measures erythrocyte mean corpuscular volume (MCV) variability (anisocytosis), has been linked to early mortality in many diseases and in older adults through unknown mechanisms. Hypoxic stress has been proposed as a potential mechanism. However, experimental models to investigate the link between increased RDW and reduced survival are lacking. Here, we show that lifelong hypobaric hypoxia (~10% O_2_) increased erythrocyte numbers, hemoglobin, and RDW, while reducing longevity in male mice. Compound heterozygous knockout (hKO) mutations in succinate dehydrogenase (Sdh; mitochondrial complex II) genes *Sdhb*, *Sdhc*, and *Sdhd* reduced Sdh subunit protein levels, reduced RDW, and increased healthy life span compared with WT mice in chronic hypoxia. RDW-SD, a direct measure of MCV variability, and the SD of MCV showed the most statistically significant reductions in Sdh hKO mice. Tissue metabolomic profiling of 147 common metabolites showed the largest increase in succinate with elevated succinate/fumarate and succinate/oxoglutarate (2-ketoglutarate) ratios in Sdh hKO mice. These results demonstrate that mitochondrial complex II level is an underlying determinant of both RDW and healthy life span in hypoxia and suggest that therapeutic targeting of Sdh might reduce high RDW–associated clinical mortality in hypoxic diseases.

## Graphical abstract

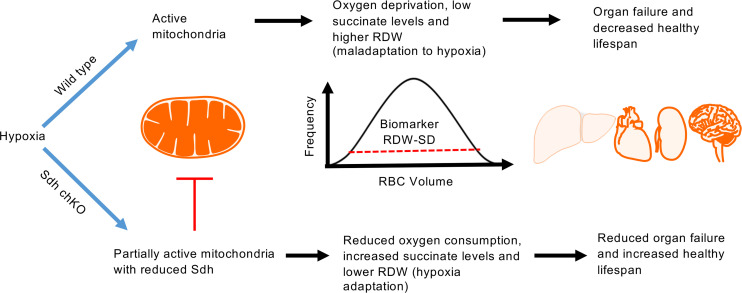

## Introduction

Erythrocyte anisocytosis refers to increased variation in red blood cell (RBC) volume and is measured by red cell distribution width (RDW) in routine complete blood count (CBC) analysis. RDW is often reported as a coefficient of variation (RDW-CV) of erythrocyte mean corpuscular volume (MCV) in an RBC volume distribution curve. RDW-CV is calculated by dividing the standard deviation (1SD-RDW) by MCV, multiplied by 100. RDW-SD is a direct measure of anisocytosis that reports the MCV variation at 20% frequency level (1). RDW increases in the healthy aging population (2).

RDW along with MCV is traditionally used in the differential diagnosis of anemia. In recent years, however, high RDW has been associated with increased mortality in acute and chronic diseases as well as in middle-aged and older individuals without disease (3, 4). The association has been observed in a growing list of clinical conditions, including heart failure (5, 6), myocardial infarction (7), peripheral artery disease (8), cancer (9), pulmonary hypertension (10), acute pulmonary embolism (11), community-acquired pneumonia (12, 13), SARS-CoV-2 infection (14), chronic obstructive pulmonary disease (15, 16), acute respiratory distress syndrome (17), acute cerebral infarction and stroke (18, 19), intensive care unit and trauma patients (20–22), hip fracture (23), sepsis and septic shock (24), Gram-negative bacteremia (25), acute pancreatitis (26), hemodialysis (27), and kidney transplant receivers (28). The underlying mechanisms that contribute to increased mortality in the context of increased RDW are unknown.

Although anisocytosis is a physiologic response to anemia, the association with mortality remains significant in nonanemic individuals (7, 29–34) and becomes even stronger in nonanemic individuals than in anemic ones in meta-analysis (35). A correlation between inflammatory markers and anisocytosis has been documented, which raises the hypothesis that the effect of RDW on mortality may be mediated by systemic inflammation (36). However, the association of anisocytosis with mortality and disease remains statistically significant even in patients with low C-reactive protein levels, a marker of inflammation (4, 37, 38). It has been hypothesized that RBCs with increased size variation may have reduced deformability that impairs microcirculatory blood flow, though contrasting results were reported on the impact of increased RDW on RBC deformation (39, 40). Furthermore, certain anemias cause marked anisocytosis without significantly increasing the mortality risk. For example, dietary iron deficiency anemia is attributed to about 0.08 deaths per 100,000 (41). These considerations collectively suggest that the mortality risk associated with anisocytosis cannot be readily explained by anemia, inflammation, or RBC physicochemical characteristics.

Increased anisocytosis may reflect a fundamental cellular pathology that predisposes to mortality regardless of the specific clinical condition. Yčas et al. analyzed over 2 million medical claims and concluded that RDW indicates systemic hypoxic load, especially in pulmonary and cardiac conditions (42). High RDW correlates with severity and poor survival in chronic obstructive pulmonary disease (15, 16, 43, 44) as well as with lung function in healthy individuals (45). A recent analysis of RDW in 121,530 nonanemic individuals with a medical condition revealed the strongest associations with pulmonary hypertension, chronic pulmonary heart disease, and congestive heart failure, which all have a pathophysiologic link to hypoxia (46). Similarly, both hypoxemia and high RDW have been linked to mortality risk in patients with COVID-19 (14, 47). Hypoxia triggers the production of RBC precursor reticulocytes from bone marrow through the operation of prolyl hydroxylase/HIF pathway that regulates erythropoietin production (48). Since reticulocytes are larger than mature RBCs, anisocytosis ensues. Thus, systemic hypoxia appears to be a biologically plausible stress factor that might explain the association between anisocytosis and mortality. However, experimental evidence for this hypothesis is lacking.

In this study, we report on the impact of chronic hypobaric hypoxia on RBC parameters and healthy life span in succinate dehydrogenase (Sdh) heterozygous and WT control male mice. In humans, heterozygous germline SDH subunit mutations predispose to paraganglioma (PGL) and pheochromocytoma tumors (49). Hereditary PGL tumors caused by *SDHD* mutations often develop in the carotid body (CB) in the neck (50) and mimic the sporadic CB PGLs caused by chronic hypoxic stimulation of high altitudes (51). Higher altitude increases the severity of hereditary PGL tumors (52, 53). Gene expression profiling studies in SDH PGLs show persistent activation of hypoxia-induced genes in normoxic conditions (pseudohypoxia) (54). These results collectively suggest that SDH mutations predispose to PGL tumors by constitutively activating the hypoxia-sensing/signaling pathways in paraganglionic tissues.

Sdh mouse models show that while homozygous deficiency of a subunit is incompatible with normal life and development, heterozygous mutations do not cause PGL tumors (55–57). Here, we present evidence that chronic hypoxic stimulation also fails to develop PGL tumors in Sdh mice. Unexpectedly, we find that mice with partial Sdh deficiency show reduced RDW and increased healthy life span relative to control mice in chronic hypoxia, revealing a mechanism contributing to the association between high RDW and mortality.

## Results

### Sdh knockout mice.

**Figure 1:**
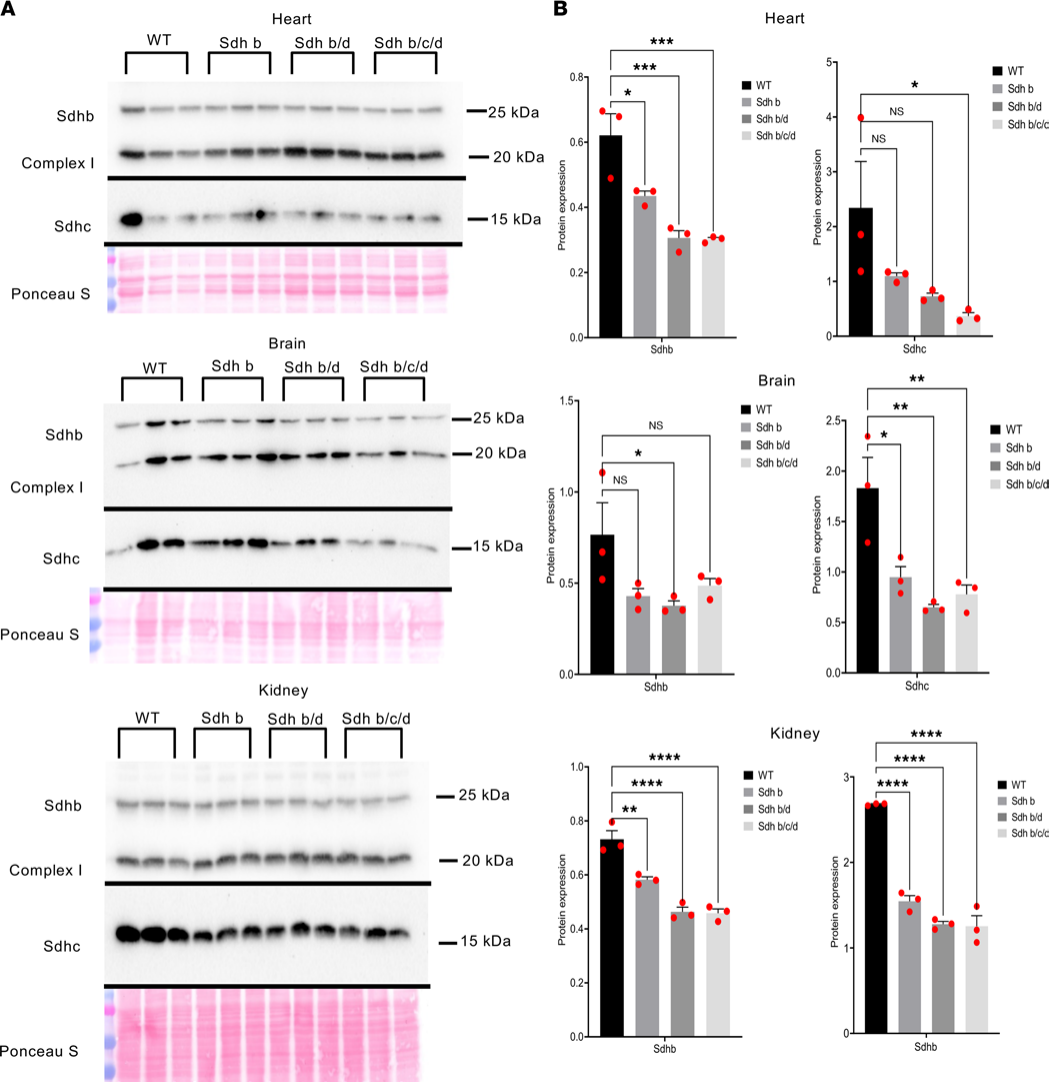
Protein levels of SDHs decrease in Sdh hKO mice. (**A**) Western blots of Sdhb, Sdhc, and complex I protein NADH:ubiquinone oxidoreductase subunit B8 (NDUFB8) from heart, kidney, and brain tissues from WT (*n* = 3), Sdhb (*n* = 3), Sdhb/d (*n* = 3), and Sdhb/c/d (*n* = 3) mice. Ponceau S staining was used as a loading control. The experiment is conducted once. (**B**) Quantification of bands’ intensity as a measurement of protein levels from heart, kidney, and brain tissues from WT (*n* = 3), Sdhb (*n* = 3), Sdhb/d (*n* = 3), and Sdhb/c/d (*n* = 3) mice. Sdhb and Sdhc were normalized to complex I NDUFB8. *P* values are calculated by ordinary 1-way ANOVA, (*P < 0.05, **P < 0.01, ***P < 0.001, ****P < 0.0001). Data represent mean ± SEM.

The experimental mice were derived by crossing the 3 previously described original strains each containing a heterozygous knockout (hKO) mutation in *Sdhb*, *Sdhc*, or *Sdhd* (see Methods) (58). Genotypes were determined by gene KO-specific PCR amplifications from tail DNA. To confirm that hKOs reduce complex II protein levels, we performed Western blot analysis in WT (*n* = 3), *Sdhb* single-hKO (*n* = 3), *Sdhb/c* double-hKO (*n* = 3), and *Sdhb/c/d* triple-hKO (*n* = 3) male mice using heart, kidney, and brain tissues (Figure 1A and Supplemental Figure 1; supplemental material available online with this article; https://doi.org/10.1172/jci.insight.158737DS1). We first evaluated linearity in dose-response relationship in Western blots (Supplemental Figure 2). Sdhb and Sdhc protein levels quantified against the control complex I protein NDUFB8 showed statistically significant reductions in single- or compound-hKO mice (Figure 1B). Notably, Sdhc protein levels were also decreased in Sdhb and Sdhb/d mice in kidney and brain. Also, Sdhb protein levels were lower in Sdhb/d and Sdhb/c/d than in Sdhb mice in heart and kidney. These results suggest that individual subunit levels decreased further by reductions in other subunits, possibly through degradation of the unincorporated proteins into complex II. These findings are consistent with the observations that immunohistochemical loss of *SDHB* in PGLs can occur with inactivating mutations in *SDHA*, *SDHB*, *SDHC*, *SDHD*, or *SDHAF2* subunit genes (59).

### Sdh hKO mice do not develop tumors under chronic hypoxia.

The development of PGLs or other tumors was prospectively examined in 3 sequentially tested groups of male mice exposed to lifelong hypoxia (~10% O_2_). Group 1 had Sdh double hKO of *Sdhb/c*, whereas groups 2 and 3 had Sdh triple hKO of *Sdhb/c/d* (Table 1). The initial goal was to determine whether compound heterozygosity in Sdh predisposes to PGL tumor development under chronic hypoxia. Noninvasive magnetic resonance imaging (MRI) analysis of hKO *Sdhb/c* (mouse 145) and WT control (mouse 197) mice after about 7 months of chronic hypoxia exposure showed no radiologic evidence of tumor development in either genotype (Supplemental Figure 3). Gross and microscopic examination of the hypoxia-exposed mice confirmed lack of tumor development or vascular pathology, such as intimal thickening or plexiform lesions in lung or pheochromocytoma development in adrenal gland (Supplemental Figure 4). Thus, although hypobaric hypoxia of high altitudes has been shown to promote development of sporadic PGLs in humans (60), we found no evidence of Sdh-related PGL tumor development in mice in normoxia or following exposure to chronic hypoxia.

### Sdh hKO mice survive longer under chronic hypoxia.

**Figure 2:**
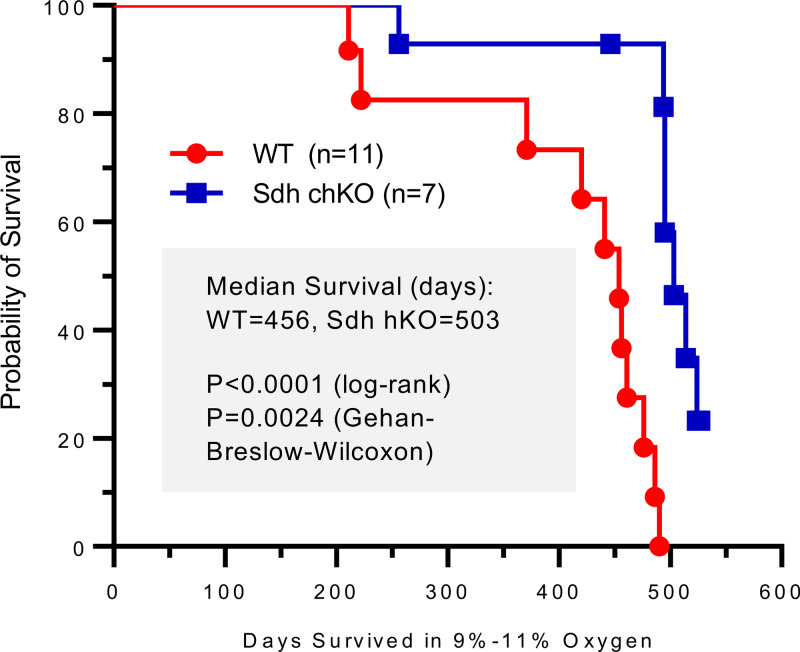
Kaplan-Meier healthy life span analysis of WT and Sdh hKO male mice from 3 groups. The curve differences (*n* = 15 WT and *n* = 15 compound hKO, chKO) are statistically significant by log-rank (Mantel-Cox) and Gehan-Breslow-Wilcoxon tests. Healthy life span is measured by the total number of days until spontaneous death or the development of morbidities that require euthanasia in chronic hypoxia. The depicted curve includes all mice that died spontaneously or were euthanized due to morbidity (e.g., all events described in Table 1). The curve excludes points for 4 WT, which include M197 and 3 mice from experiment 3 (Table 1), and 8 hKO, which include M145, M515, M536, and 5 mice from experiment 3 (Table 1). These mice are censored since they did not complete the life span endpoints.

We observed 3 sequential groups of hKO and WT control male mice until spontaneous death or development of morbidities that required euthanasia (i.e., healthy life span). WT control mice were of similar age and selected from the same or closely related litters. Healthy life spans of mice in chronic hypoxia (median 503 days for Sdh hKO and 456 days for WT control) were substantially lower than those of the parental B6 mice living in room conditions (~2.5 years). However, we found that Sdh hKO mice survived longer than WT control mice in each of the 3 experimental groups (Table 1). When data from the 3 groups were combined, the life span differences between Sdh hKO and WT mice were statistically significant (*P* < 0.0001 by log-rank [Mantel-Cox] test and *P* = 0.0024 by Gehan-Breslow-Wilcoxon test) (Figure 2). The rate of hypoxic death/moribund conditions in WT mice, estimated by hazard ratio, was 11.39-fold (95% CI of 3.419 to 37.95) and 4.65-fold (95% CI of 1.606 to 13.47) higher than Sdh hKO mice by Mantel-Haenszel and log-rank methods, respectively. Four WT mice and 1 *Sdhb/c* mice were euthanized based on institutional guidelines due to the development of morbid conditions. The survival difference between Sdh hKO (*n* = 6) and WT (*n* = 7) mice was statistically significant even when the mice euthanized for moribund conditions were excluded from the analysis (*P* = 0.0037 log-rank [Mantel-Cox] test and *P* = 0.0304 Gehan-Breslow-Wilcoxon test).

Necropsy of mice revealed no specific causes to explain early death or the development of moribund conditions but showed congestion and enlargement of spleen and heart, which are expected under chronic hypoxia. Chronic hypoxia is associated with the development of pulmonary hypertension and right ventricular hypertrophy. We assessed right ventricular hypertrophy by Fulton index in experimental group 2 and found no statistically significant differences between Sdh hKO and WT control mice (Supplemental Figure 5). This result suggests that pulmonary hypertension differences probably do not explain the differential survival between the Sdh hKO and WT mice.

### Sdh hKO mice show evidence of reduced RBC regeneration and lower RDW.

**Figure 3:**
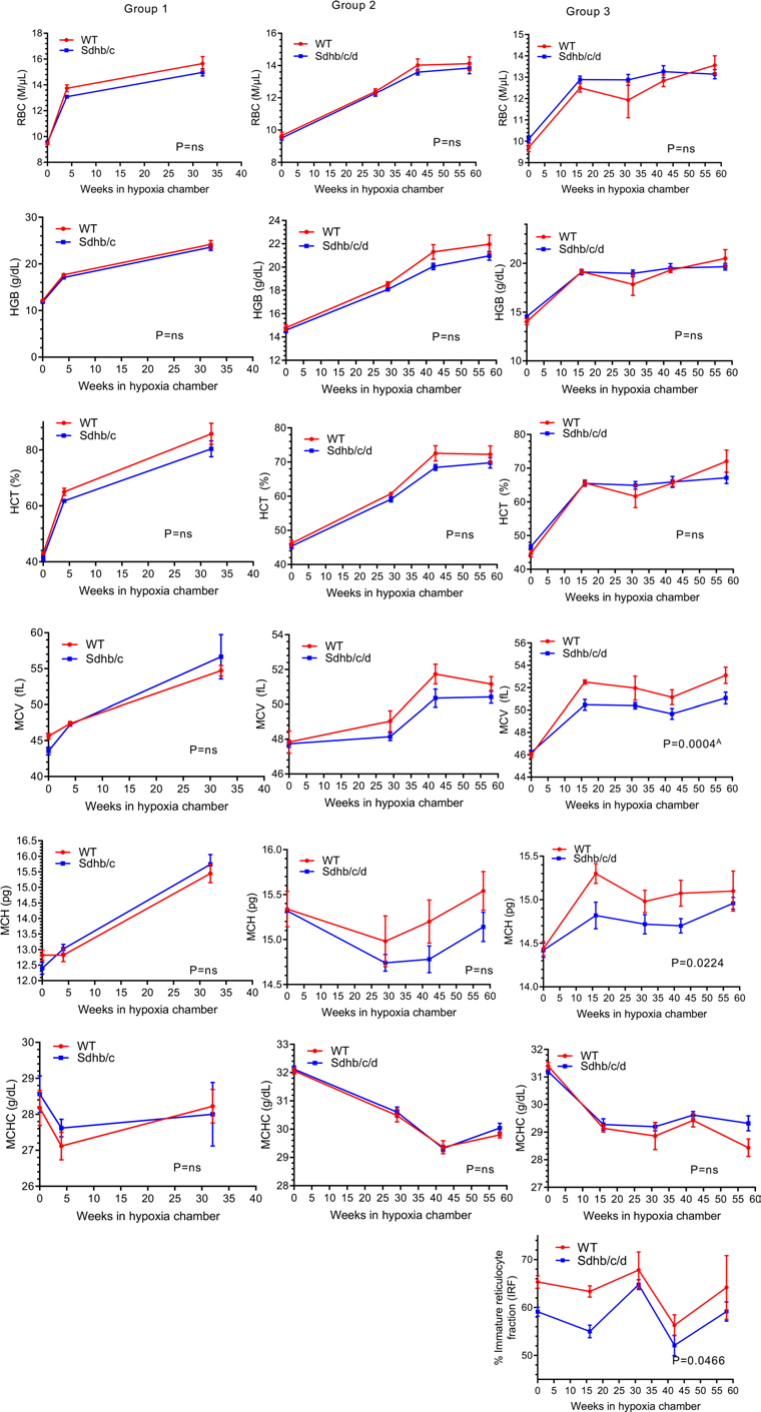
RBC, hemoglobin, hematocrit, mean corpuscle volume, mean corpuscle hemoglobin, mean corpuscle hemoglobin concentration, and immature reticulocyte fraction in Sdh hKO (Sdhb/c in group 1 and Sdhb/c/d in groups 2 and 3) and WT control mice under chronic hypoxia. RBC numbers, hemoglobin (HGB) and hematocrit (HCT), mean corpuscle volume (MCV), mean corpuscle hemoglobin (MCH), mean corpuscle hemoglobin concentration (MCHC), and immature reticulocyte fraction (IRF) are shown. Each time point contains 3–5 male mice and shows mean and SEM. *P* values are calculated by 2-way ANOVA using time and genotype as independent variables. ^A^*P* value remains significant (less than 0.05) after adjustment for multiple comparisons by Holm-Šídák method (α: 0.05). The missing parameter IRF in groups 1 and 2 was not available in earlier CBC outputs. HGB results of groups 1 and 2 were previously shown in Sharma et al. (58) and included here for comprehensive analysis.

**Figure 4:**
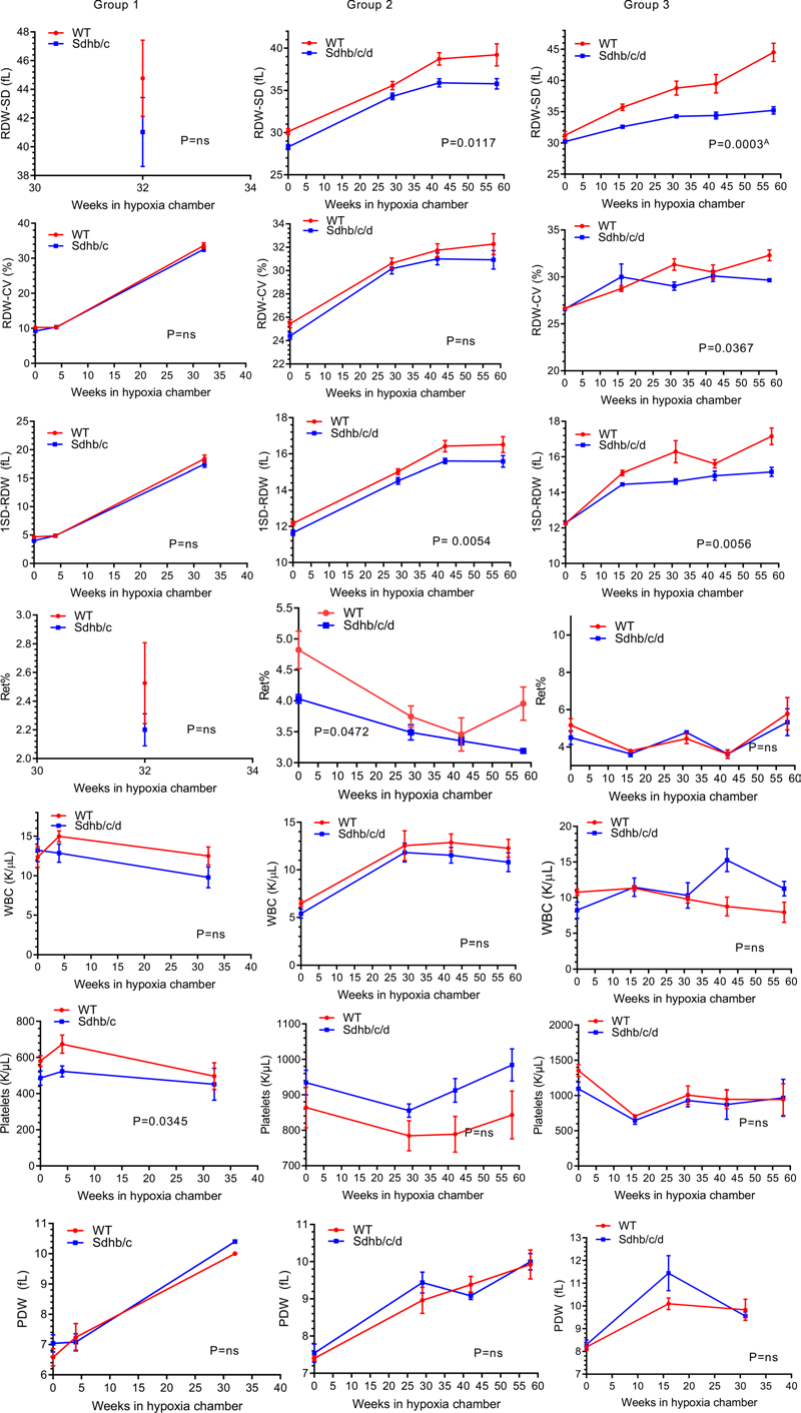
RDW-SD, RDW-CV, 1SD-RDW, reticulocyte percentage, white blood cells, platelets, and platelet distribution width in Sdh hKO (Sdhb/c in group 1 and Sdhb/c/d in groups 2 and 3) and WT control mice under chronic hypoxia. Measures of RBC size variation including RDW-SD, RDW-CV and 1SD-RDW, reticulocyte percentage (Ret%), white blood cells (WBCs), platelets, and platelet distribution width (PDW) are shown. Each time point contains 3–5 male mice and shows mean and SEM. *P* values are calculated by 2-way ANOVA using time and genotype as independent variables. ^A^*P* value remains significant (less than 0.05) after adjustment for multiple comparisons by Holm-Šídák method (α: 0.05). The missing parameters in group 1 (RDW-SD, Ret%) were not available in earlier CBC outputs. The missing parameter IRF in groups 1 and 2 was not available in earlier CBC outputs.

To examine whether erythrocyte numbers could explain the survival differences between the 2 genotypes, we analyzed CBC variables from 3 groups of Sdh hKO male mice and WT controls using 2-way ANOVA test. No statistically significant differences were observed in RBC numbers or hemoglobin (HGB) levels in any group (Figure 3). Reduced HCT (Figure 3), MCV, MCH (Figure 3), RDW-CV (Figure 4), reticulocyte percentage (Figure 4), and IRF (Figure 3) were observed in Sdh hKO mice in 1 of 3 groups. The most statistically significant differences between the genotypes were observed in direct measures of RBC size variability, namely RDW-SD and 1SD-RDW, which showed a reduction in Sdh hKO mice in 2 of 3 groups (Figure 4).

Analysis of the combined data from all 3 groups showed the most statistically significant differences in RDW-SD, RDW-CV, 1SD-RDW, and IRF both in normoxia and in hypoxia, with lower values observed in Sdh hKO mice relative to WT control mice (Table 2). Borderline statistically significant differences were seen in HCT and MCV in hypoxia and Ret% in normoxia. No statistically significant differences were seen in the numbers of WBCs and platelets and in PDW between Sdh hKO and WT mice in normoxia or hypoxia (Figure 4 and Table 2). Collectively, the differences in RBC parameters reveal blunted rate of erythropoietic activity and erythrocyte regeneration by partial loss of Sdh, especially in hypoxia.

### Metabolomic analysis shows increased succinate levels and succinate/fumarate and succinate/oxoglutarate ratios in Sdh hKO mice.

**Figure 5:**
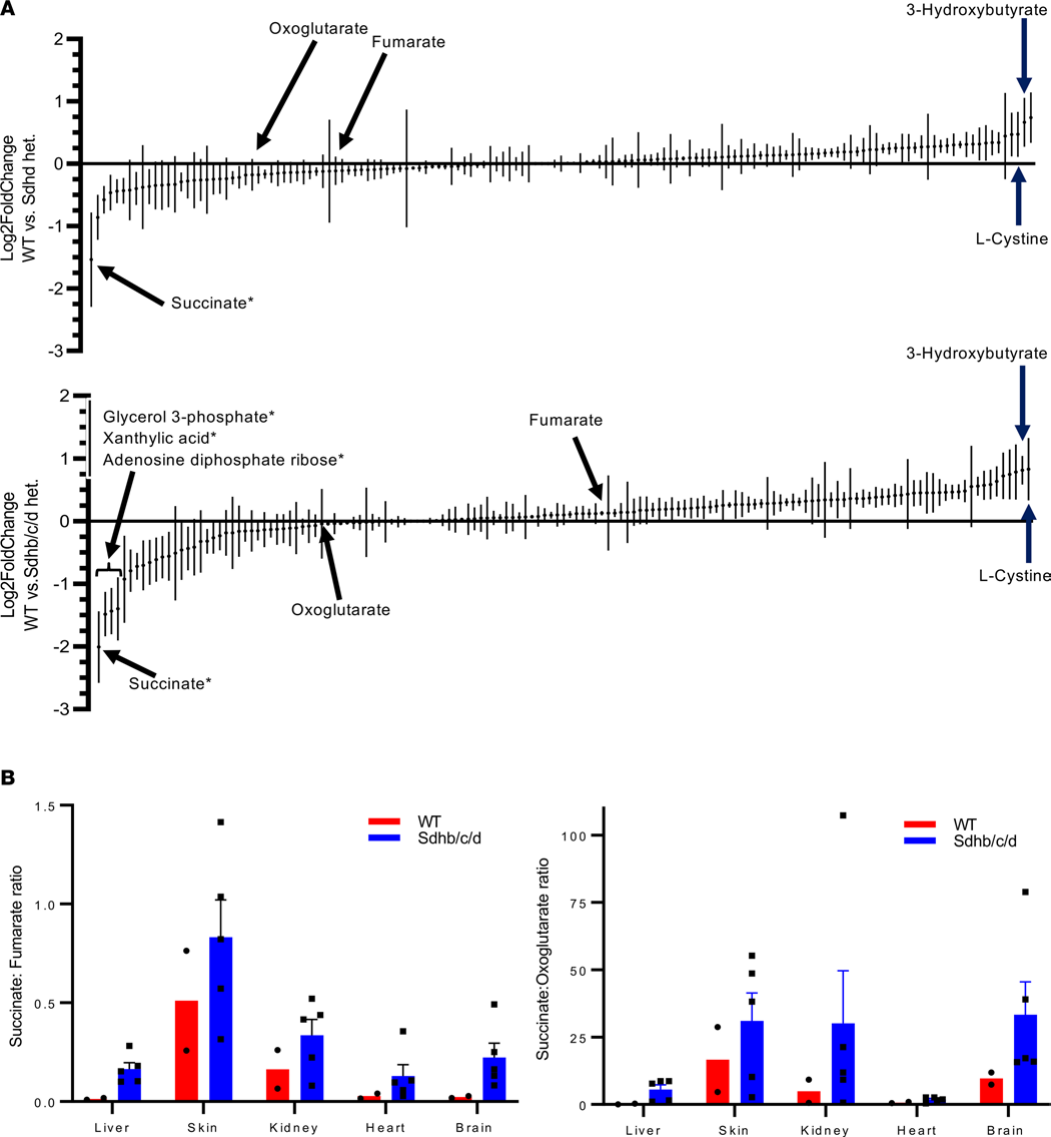
Metabolite profiling in WT versus Sdh hKO mice under normoxic conditions. (**A**) Average fold changes (log2FoldChange) of liver, heart, kidney, skin, and brain in 147 metabolites are ranked. Both Sdhd single-hKO (*n* = 3 mice) and Sdhb/c/d triple-hKO (*n* = 5 mice) tissues showed the highest increase in succinate, and the highest overlapping decreases in 3-hydroxybutyrate and l-cystine, relative to WT control mice (*n* = 2). A stringent outlier analysis by ROUT method (*Q* = 0.1%) detected only 4 outlier metabolites, marked by asterisks. (**B**) The ratios of succinate to fumarate and oxoglutarate were increased in Sdhb/c/d-hKO mice relative to WT. Data represent mean ± SEM.

To examine metabolic differences between Sdh hKO mice (*n* = 3 Sdhd and *n* = 5 Sdhb/c/d) and WT (*n* = 2) mice, we obtained metabolic profiles of liver, skin, kidney, heart, and brain by quantifying 147 common metabolites (Supplemental Table 2). A principal component analysis showed distinct clustering among each tissue type, with the heart samples and the brain samples grouping closely together, irrespective of the genotype (Supplemental Figure 6). We ranked the metabolites by the average of their log_2_ fold change between genotypes across 5 tissues and examined the extreme outlier metabolites that did not fit to a Gaussian distribution using ROUT method and FDR of *Q* = 0.1% (61). Succinate was the only outlier metabolite identified in both Sdhd and Sdhb/c/d hKO mice (Figure 5A). Glycerol 3-phosphate, xanthylic acid, and adenosine diphosphate ribose were also identified as increased outliers in Sdhb/c/d hKO mice but not in Sdhd mice. Succinate increased by 2.9-fold and 4.02-fold in Sdhd and Sdhb/c/d hKO mice, respectively, leading to increased ratios of succinate to fumarate and oxoglutarate (2-ketoglutarate) (Figure 5B). The most decreased metabolites commonly detected in both hKO genotypes were 3-hydroxybutyrate and l-cystine, which decreased by more than 1.5-fold but less than 2-fold (Figure 5A). Neither metabolite was identified as an outlier by the ROUT method.

## Discussion

In this study, we show that hKO mutations in Sdh genes prolong healthy life span by approximately 10% under chronic hypoxia and reduce multiple measures of RBC anisocytosis, including RDW-SD, RDW-CV, and 1SD-RDW. Other parameters related to RBC regeneration, including IRF, HCT, and MCV, also show evidence of reductions in Sdh hKO mice compared with WT. The lack of statistically significant differences in RBC numbers or HGB suggests that Sdh regulates the rate of hypoxia-dependent RBC regeneration but not the total RBC or HGB levels. We find no evidence of PGL tumor development in mice even with chronic lifelong hypoxia exposure, in agreement with a recent study (57). These findings collectively show a potentially previously unrecognized role for Sdh in regulation of erythroid regeneration both in normoxia and in hypoxia. To our knowledge, this is also the first mammalian study showing a hypoxia survival benefit upon partial constitutional loss of Sdh. Our findings parallel those observed in other organisms.

Studies in *Ascaris suum*, a helminthic parasite, show that Sdh is active in spore forms that respire atmospheric O_2_ but not in the adult forms, which live in the hypoxic environment of the host intestine. The adult parasite instead uses fumarate reductase (Frd), which catalyzes the reverse reaction of Sdh (62). A hypoxic switch in Sdh genes controls respiration in *Mycobacterium tuberculosis* (63). ATP-producing eukaryotic mitochondria use Frd rather than Sdh under limited O_2_ conditions (64). Flies resistant to hypoxia have reduced complex II activity levels compared with control flies (65). Anoxic environments (N_2_ or CO_2_) lead to decreased transcript expression of 3 of the 4 SDH subunit genes, through promoter methylation in maize (66). Furthermore, suppressing mitochondrial respiration was found to promote hypoxia tolerance in fetal growth plate (67). Our findings combined with these studies suggest that inhibition of Sdh is a universal theme in organismal adaptation to hypoxia/anoxia across diverse organisms, including mammals.

Identification of the molecular mechanisms linking reduced Sdh to organismal tolerance to hypoxia requires further studies. There is, however, existing evidence that loss of SDH triggers hypoxia adaptation pathways in human PGL tumors (54). Several studies have shown increased succinate/fumarate and succinate/oxoglutarate ratios in SDH-mutated PGL tumors. Increased succinate relative to oxoglutarate inhibits α-ketoglutarate–dependent enzymes, including HIF prolyl hydroxylases (68), Jumonji domain histone demethylases (69), and TET family of 5-methyldeoxycytosine hydroxylases (70), contributing to pseudohypoxia and hypermethylation in PGL tumors. We also find increased succinate/fumarate and succinate/oxoglutarate ratios in tissues from Sdh hKO mice, even though global tissue-specific metabolite profiles were not drastically altered by partial Sdh loss. In contrast to expectations that Sdh partial loss could further enhance erythropoiesis by succinate-mediated inhibition of HIF prolyl hydroxylases, leading to HIFα stabilization, we see evidence of *reduced* erythropoiesis. This finding suggests that increased intracellular oxygen availability upon partial loss of Sdh plays a more dominant role in HIFα regulation by promoting HIF prolyl hydroxylase activity than the inhibitory impact of increased succinate levels. It is conceivable that alterations in succinate, 3-hydroxybutyrate, and l-cystine or other metabolite levels contribute to systemic hypoxia tolerance and increased healthy life span in Sdh hKO mice. The *SDHD* gene is subject to maternal imprinting (inactivation) in hypoxia-sensitive CB chief cells, because only a paternal transmission, but not maternal transmission, of the mutated *SDHD* gene predisposes to PGL tumors. Thus, partial loss of SDH activity by genomic imprinting might facilitate hypoxia sensing and/or adaptation in CB cells (71). We recently showed that pharmacologic inhibition of complex II by atpenin A5 triggers hypoxic gene expression and RNA editing by APOBEC3A and APOBEC3G cytidine deaminases independently of HIF1 in monocytes and natural killer (NK) cells, respectively (58, 72). The *SDHB* and *SDHA* genes acquire nonsense/missense RNA editing by APOBEC3A in monocytes subjected to cellular crowding and hypoxia (73, 74). RNA editing by APOBEC3G in NK cells is induced by cellular crowding and hypoxia and promotes Warburg-like metabolic remodeling by suppressing O_2_ consumption relative to glycolysis (72). It is conceivable that the inhibition of Sdh in mice activates similar HIF-independent hypoxia adaptation pathways, including gene expression, RNA editing, and possibly other adaptive pathways that remain to be discovered.

Importantly, our findings suggest a mitochondrial basis for the association between high RDW and mortality observed in many diseases. Previous research has established that inhibition of mitochondrial respiration antagonizes the hypoxic stabilization of HIFα (75, 76), the key molecular event driving the synthesis of erythropoietin that stimulates RBC regeneration in bone marrow. Pharmacologic inhibition of complex II by atpenin A5 reduces the stabilization of HIFα in cancer cell lines in hypoxia and reduces baseline O_2_ consumption (58, 77). Atpenin A5 is a highly potent complex II inhibitor of ubiquinone binding at the interface of Sdhb, Sdhc, and Sdhd subunits (78, 79). Therefore, we suggest that partial loss of Sdh in hKO mice reduces mitochondrial O_2_ consumption and leads to blunted HIF-mediated RBC regeneration and RDW in hypoxia.

Jain et al. (80) found that hypoxia and inhibition of Von Hippel–Lindau protein, which triggers HIF-mediated cellular hypoxia response, promote survival in a genetic mouse model with mitochondrial respiratory defect, and in cell culture and zebrafish models, respectively. Although the exact mechanisms are unclear, normal O_2_ levels seem detrimental when mitochondrial respiration is impaired. Our model suggests that the opposite is also true: reduced O_2_ levels are detrimental when the mitochondrial respiration is intact. Therefore, it appears that optimum organismal survival requires a balance between mitochondrial activity levels and O_2_ abundance and that Sdh levels play an important role in hypoxic survival.

Our study has certain limitations, including the lack of a specific disease model in which high RDW has been associated with early mortality in clinical studies and the indeterminate causes of death in hypoxic mice. Also, these findings were obtained in male mice only and remain to be extended to female mice. Further studies are required to close these knowledge gaps in the future.

**Figure 6:**
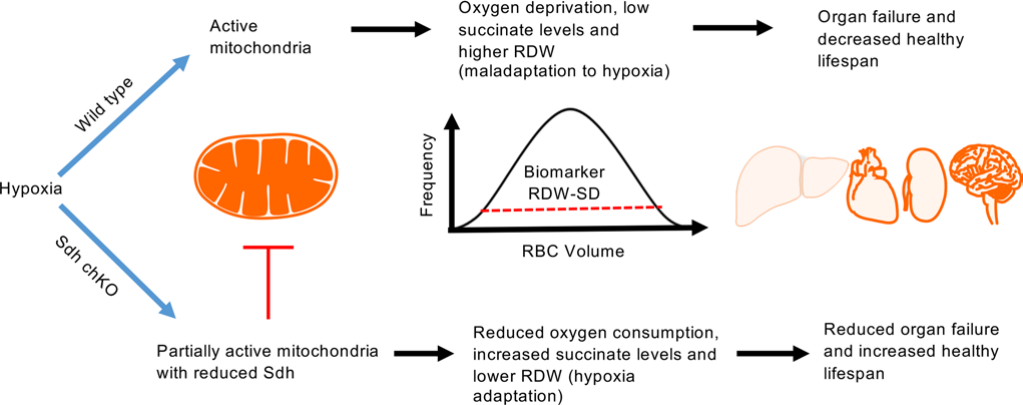
A mitochondrial basis for the association between high RDW and mortality in hypoxia. When oxygen is limited, fully active mitochondria exhaust the remaining oxygen, leading to oxygen deprivation, erythrocyte regeneration, high RDW, compromised cellular viability, organ failure, and mortality. Inhibition of Sdh reduces oxygen consumption and RDW levels and triggers cellular hypoxia adaptation pathways leading to improved survival.

In summary, our findings show that Sdh plays an unanticipated role in regenerative erythrocyte anisocytosis and organismal life span in mice under chronic hypoxia. These results support a model that upon systemic hypoxia, mitochondrial O_2_ consumption depletes cellular O_2_, leading to cellular injury, organ failure, and death while increasing RDW through HIF-dependent erythropoiesis. Suppressing Sdh reduces O_2_ consumption, mitigates cellular hypoxia, blunts RDW, and triggers HIF-independent hypoxia adaptation pathways to promote organismal tolerance to chronic hypoxia (Figure 6). Direct testing of tissue O_2_ consumption rates in Sdh mice remains to be performed, although we and others reported diminished mitochondrial O_2_ consumption upon Sdh inhibition by atpenin A5 in mammalian cells (58, 77). We hypothesize that high RDW is merely a surrogate biomarker for cellular hypoxia, which is controlled in part by mitochondrial O_2_ consumption, and that hypoxia is the ultimate driver of cell death, organ failure, and mortality. Therefore, therapeutic targeting of Sdh may reduce high RDW–associated mortality in hypoxic diseases by enhancing systemic adaptation to hypoxia.

## Methods

### Sdh KO mice.

*Sdhb* and *Sdhc* hKO mice were created in The Jackson Laboratory in B6/129P2 background and described as B6.129P2-Sdhb<Gt(AP0532)Wtsi>/Cx and B6.129P2-Sdhc<Gt(BA0521)Wtsi>/Cx. (58). The *Sdhd* KO mouse (81) was rederived into C57BL/6J background at Roswell Park Comprehensive Cancer Center (RPCCC) transgenic facilities using frozen sperm (mfd Diagnostics). As previously reported, homozygous mutations in any subunit are nonviable, but compound *Sdhb/Sdhc* double heterozygous and *Sdhb/Sdhc/Sdhd* triple heterozygous KO mice are viable (58). Since each gene is located on a different mouse chromosome, the KO alleles segregate independently and give the expected numbers of each viable genotype upon crossing the Sdh hKO mice. Control WT mice were also derived from crosses of Sdh hKO mice. WT controls in hypoxia experiments were either littermates or from closely related litters. Genotyping was performed in tail tips at the RPCCC transgenic facility as described (58). Genotypes of the mice in the hypoxia chamber were confirmed by repeat testing.

### Hypoxia exposure.

Mice were exposed to chronic hypobaric hypoxia in a custom-made hypoxia chamber (Case Western Reserve University Design Fabrication Center, Cleveland, Ohio, USA) that operates via house vacuum and accommodates 2 standard mouse cages (5 mice per cage), as previously described (58). Mice were initially subjected to mild hypoxia (~14% O_2_) for about 1 week for acclimatization. For chronic exposure, the oxygen concentration was about 10% with a range of 9%–11%. Oxygen percentage was continuously monitored by an O_2_ sensor. Hypoxia exposure experiments involved 5 compound heterozygous and 5 WT control mice (~10 weeks of age), with each genotypic group placed in a different cage. Mice were daily observed and briefly removed from the chamber twice a week for cage cleaning.

Mice remained in the hypoxia chamber until spontaneous death or the development of morbid conditions, as assessed during cage cleaning, that required euthanasia in accordance with RPCCC animal care guidelines and the approved IACUC protocol. Examples of morbid conditions included limited or absent movement, hunched posture, labored breathing, sunken eyes, shaking, and development of rectal prolapse. All decisions for euthanasia due to morbid status were made in accordance with approved IACUC guidelines. Organs were grossly examined during necropsy. Tissues were collected upon spontaneous death and euthanasia.

### Peripheral blood analysis.

Body weights were measured, and blood was collected for CBC analysis. Blood (~0.2 mL) was collected into EDTA tubes by retro-orbital bleeding at baseline and subsequent time points. Alternate eyes were used for a maximum of 2 times per eye. Additional bleeding was performed by mandibular venipuncture. CBCs were analyzed via automated cell counters Hemagen HC5 (first 2 time points in group 1) or ProCyte Dx (the third time point in group 1, group 2, and group 3) hematology analyzers available at Roswell Laboratory Animal Shared Resources. Certain RBC parameters, such as RDW-SD, Ret%, and IRF, were not reported by Hemagen HC5 counter. The RBC parameter values examined in this study are direct outputs of the analyzers, except 1SD-RDW, which is derived as (RDW-CV × MCV)/100.

### Subcellular fractionation.

Mitochondria were isolated from mouse tissues by homogenization and centrifugation. Briefly, fresh-frozen tissues were thawed in homogenization buffer (20 mM HEPES, pH 7.4; 10 mM KCl; 1.5 mM MgCl_2_; 1 mM EDTA; 1 mM EGTA; 250 mM sucrose) supplemented with freshly added 1× protease inhibitor cocktail (Thermo Fisher Scientific, catalog 78440) and 1 mM DTT. A total of 1 mL of homogenization buffer was used per 100 mg tissue. Tissues were chopped as much as possible using fine-pointed scissors in the homogenization buffer and incubated for 30 minutes in ice with intermittent vortexing. Tissues were then homogenized using a dounce homogenizer (~35–40 strokes using loose pestle) and precleared of debris using centrifugation at 1,000*g* for 10 minutes. Supernatant was collected in a new tube and centrifuged at 13,500*g* for 20 minutes to obtain a mitochondrial pellet. All centrifugation steps were performed at 4°C. Supernatant was collected as cytosolic fraction, and mitochondrial pellets were washed 4 times with homogenization buffer. Mitochondrial pellets were lysed in RIPA buffer and assayed as the mitochondrial fraction.

### Western blotting.

Cytoplasmic and mitochondrial lysates were mixed in Laemmli buffer; denaturized for 5 minutes at 95°C and 55°C, respectively; and run on homemade 15% SDS-PAGE. Proteins were then transferred to nitrocellulose membranes (0.2 μm, Bio-Rad, catalog 1620112) at a constant voltage of 100 V for 70 minutes at 4°C using Mini Trans-Blot Cell (Bio-Rad). Membranes were incubated in Tris-buffered saline (TBS) with 0.1% v/v Tween-20 (MilliporeSigma) and 5% w/v nonfat dry milk (Blotting-Grade Blocker 1706404, Bio-Rad). Primary antibodies were diluted in 3% BSA in TBS with 0.1% Tween-20 and applied to the nitrocellulose membrane as follows: mouse total OXPHOS rodent antibody cocktail (Abcam, product number ab110413, 1:1000 dilution) and rabbit polyclonal anti-SDHC polyclonal antibody (Thermo Fisher Scientific, product number 14575-1-AP, 1:500 dilution). For secondary antibodies, horseradish peroxidase–conjugated donkey anti-rabbit (Thermo Fisher Scientific, catalog number 45-000-682) or goat anti-mouse (MilliporeSigma, catalog number A4416) IgG antibodies were used at 1:2,000 dilution. Pierce ECL Western Blotting Substrate (Thermo Fisher Scientific, catalog number 32106) was used for chemiluminescent detection. Signals were visualized and imaged using the ChemiDoc XRS+ System and Image Lab Software (Bio-Rad). Protein quantification was performed using ImageJ software (NIH) by measuring bands’ intensity. To evaluate linear signal-response relationship in Western blots, 1 sample from each genotype group was loaded in 3 different concentrations of each: 2.5, 5, and 10 μg. We probed for OXPHOS and Sdhc, quantified the signals, and plotted them individually for linearity. This experiment showed expected directionality in 35 of 36 signal intensity comparisons, except for Sdhc WT 2.5 versus 5 μg comparison (Supplemental Figure 2).

### MRI.

Experimental MRI examination was performed using a 4.7 T/33 cm horizontal bore magnet (GE NMR Instruments) within the Translational Imaging Shared Resource (TISR) at RPCCC. Preliminary scout images were acquired on the sagittal plane for localization and for determination of subsequent slice prescriptions. Coronal T2-weighted images were acquired using protocols previously described by us (82, 83).

### Metabolomic extraction and analysis.

Tissues (liver, kidney, heart, skin, and brain) from 10 mice (2 WT, 3 *Sdhd* single hKO, and 5 *Sdhb/c/d* triple hKO) were assayed for glycolytic and tricarboxylic acid cycle intermediates at NYU Langone’s Metabolomics Laboratory. Samples were analyzed by liquid chromatography-mass spectrometry (LC-MS) assay after scaling the metabolite extraction to a measured aliquot. Each of the 50 frozen samples was weighed on an analytical balance and then extracted in 80% methanol buffer on dry ice containing 500 nM of labeled amino acid internal standards, using a ratio of 10 mg tissue/mL extraction solution. Samples were homogenized in a BeadBlaster (Benchmark Scientific) using about 100 μL zircon beads (Research Products International) (0.5 mm) and centrifuged (21,000*g* for 3 minutes at 4°C), and then 450 μL of the supernatant was dried down by speed vacuum concentration and reconstituted in 50 μL of LC-MS–grade water. Reconstituted samples were sonicated for 2 minutes and then transferred into LC-MS vials with glass inserts.

### LC-MS/MS with the hybrid metabolomics method.

Samples were subjected to an LC-MS analysis to detect and quantify known peaks. Intensities were extracted with an in-house script with a 10 parts per million tolerance for the theoretical *m*/*z* of each metabolite and a maximum 30 second retention time window. A metabolite extraction was carried out on each sample based on a previously described method (84). The LC column was a MilliporeSigma ZIC-pHILIC (2.1 × 150 mm, 5 μm) coupled to a Dionex Ultimate 3000 system, and the column oven temperature was set to 25°C for the gradient elution. A flow rate of 100 μL/min was used with the following buffers: A) 10 mM ammonium carbonate in water, pH 9.0; and B) neat acetonitrile. The gradient profile was as follows; 80%–20% B (0–30 minutes), 20%–80% B (30–31 minutes), 80%–80% B (31–42 minutes). Injection volume was set to 2 μL for all analyses (42 minutes total run time per injection).

MS analyses were carried out by coupling the LC system to a Thermo Q Exactive HF mass spectrometer (Thermo Fisher Scientific) operating in heated electrospray ionization mode. Method duration was 30 minutes with a polarity-switching data-dependent top 5 method for both positive and negative modes. Spray voltage for both positive and negative modes was 3.5 kV, and capillary temperature was set to 320°C with a sheath gas rate of 35, auxiliary gas of 10, and max spray current of 100 μA. The full MS scan for both polarities utilized 120,000 resolution with an automatic gain control (AGC) target of 3 × 10^6^ and a maximum injection time (IT) of 100 ms, and the scan range was from 67 to 1000 *m/z*. Tandem MS spectra for both positive and negative mode used a resolution of 15,000, AGC target of 1 × 10^5^, maximum IT of 50 ms, isolation window of 0.4 *m*/*z*, isolation offset of 0.1 *m*/*z*, fixed first mass of 50 *m/z*, and 3-way multiplexed normalized collision energies of 10, 35, and 80. The minimum AGC target was 1 × 10^4^ with an intensity threshold of 2 × 10^5^. All data were acquired in profile mode.

### Relative quantification of metabolites.

The resulting Thermo RAW files were read using ReAdW.exe version 4.3.1 to enable peak detection and quantification. The centroided data were searched using an in-house python script, Skeleton version 4.0, and peak heights were extracted from the RAW files based on a previously established library of metabolite retention times and accurate masses adapted from the Whitehead Institute (84) and verified with authentic standards and/or high-resolution MS/MS spectral manually curated against the NIST14MS/MS (85) and METLIN 2017 (86) tandem mass spectral libraries. Metabolite peaks were extracted based on the theoretical *m*/*z* of the expected ion type, e.g., [M+H]^+^, with a ±5 parts per million tolerance and a ±7.5 second peak apex retention time tolerance within an initial retention time search window of ±0.5 minute across the study samples. The resulting data matrix of metabolite intensities for all samples and blank controls was processed with an in-house statistical pipeline, Metabolyze version 1.0, and final peak detection was calculated based on a signal/noise ratio of 3× compared with blank controls, with a floor of 10,000 arbitrary units. For samples where the peak intensity was lower than the blank threshold, metabolites were annotated as not detected, and the threshold value was imputed for any statistical comparisons to enable an estimate of the fold change as applicable. The resulting blank-corrected data matrix was then used for all groupwise comparisons, and 2-tailed, equal-variance *t* tests were performed with the Python SciPy (1.1.0) (87) library to test for differences and generate statistics for downstream analyses, unless otherwise specified. Any metabolite with *P* < 0.05 was considered significantly regulated (up or down). Heatmaps were generated with hierarchical clustering performed on the imputed matrix values utilizing the R library pheatmap (1.0.12) (88). Volcano plots were generated utilizing the R library Manhattanly (0.2.0).

### Statistics.

Statistical analysis and graphic presentations were performed with GraphPad Prism (Versions 7.03 and 9.2.0). *P* values of less than 0.05 (2-tailed) were considered statistically significant. Band intensities in Western blots were compared by ordinary 1-way ANOVA. Since limited preplanned comparisons were made in band intensities, corrections for multiple comparisons were not performed. CBC output values were first entered into Microsoft Excel and then imported into GraphPad. The full CBC data used in analyses are provided as Supplemental Table 1. A few extreme outlier CBC values (9 of 1393 = ~0.65%) were removed by ROUT method. The comparisons of CBC values over time between Sdh hKO and WT controls were performed by 2-way ANOVA test by using data from all available time points, including the baseline normoxic values, within each experimental group. The independent variables are time and genotype. The data are arranged so that each row represents a different time point and matched values from each mouse are stacked in a subcolumn. Two-way ANOVA tests were performed to test the main effects only (time and genotype). Since erythrocytosis is a well-known response to chronic hypoxia, we only report genotype differences. As implemented in GraphPad Prism, we used Geisser-Greenhouse correction (i.e., sphericity or equal variability of differences was not assumed). When there were no missing data, repeated measures ANOVA was used. However, since most of the analyzed data sets contained random missing values (due to loss of mice, technical errors in CBC analysis, or outlier removal), mixed effects model was used since repeated measures ANOVA requires no missing values. When the genotype comparison was made in normoxia or hypoxia, data from all time points from all 3 groups were combined, and ordinary 2-way ANOVA was used. *P* values that remained significant (less than 0.05) after adjustment for multiple comparisons by Holm-Šídák method (α: 0.05) are indicated in Figures 3 and 4 and Table 2. The survival differences were calculated by Kaplan-Meier method, where the outcome was time until death or the development of morbid conditions that required euthanasia. Outlier detections in CBC and average of log_2_ fold change in 5 tissues in metabolomics data were performed using the most stringent criteria of GraphPad’s ROUT method, which is *Q* = 0.1% (i.e., no more than 0.1% of the identified outliers are false).

### Study approval.

All experimental and mouse protocols were approved by Roswell Park Cancer Institute IACUC, Buffalo, New York, USA.

## Author contributions

BEB designed the study with contributions from DT, SS, and DRJ. AAA performed Western blot analysis and prepared Figure 1. TCR and DRJ performed the metabolomics study. DT performed mouse handling, care, identification, and breeding. DT, SS, and LC performed mouse physical evaluations, blood draws, and necropsy. MS performed MRI analysis. BEB performed the statistical analysis, prepared the remaining figures, and wrote the manuscript with contributions from AAA and DRJ. All authors reviewed the manuscript and agreed on the authorship.

## Supplementary Material

Supplemental data

Supplemental table 1

Supplemental table 2

## Figures and Tables

**Table 2:**
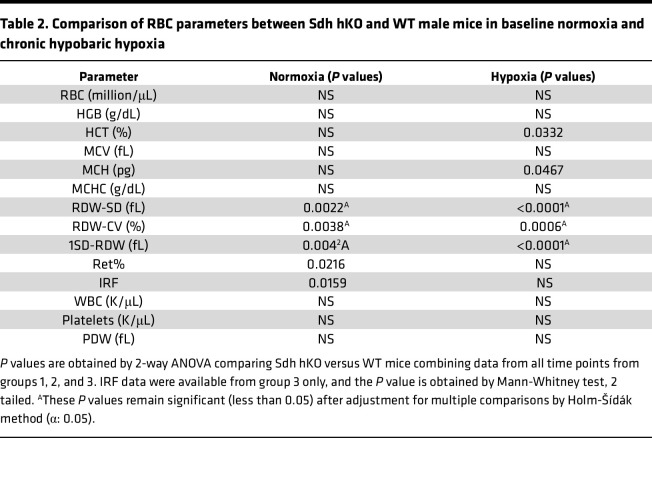
Comparison of RBC parameters between Sdh hKO and WT male mice in baseline normoxia and chronic hypobaric hypoxia

**Table 1:**
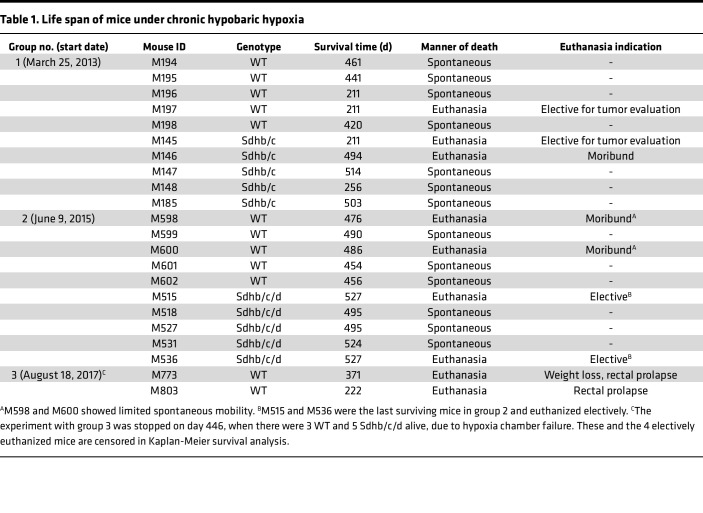
Life span of mice under chronic hypobaric hypoxia

## Version 1 07/26/2022

In-Press Preview

## Version 2 09/08/2022

Electronic publication
