# Supplementary material for: Succinate dehydrogenase inversely regulates red cell distribution width and healthy life span in chronically hypoxic mice
Source: JCI Insight. 2022 Sep 8;7(17):e158737. doi: 10.1172/jci.insight.158737 (PMC9536274; doi:10.1172/jci.insight.158737)
Supplement: Supplemental data [file jciinsight-7-158737-s067.pdf]

# Full unedited gels for Figure 1
# Kidney (Top) + Heart (Bottom)

Supplemental Figure 1

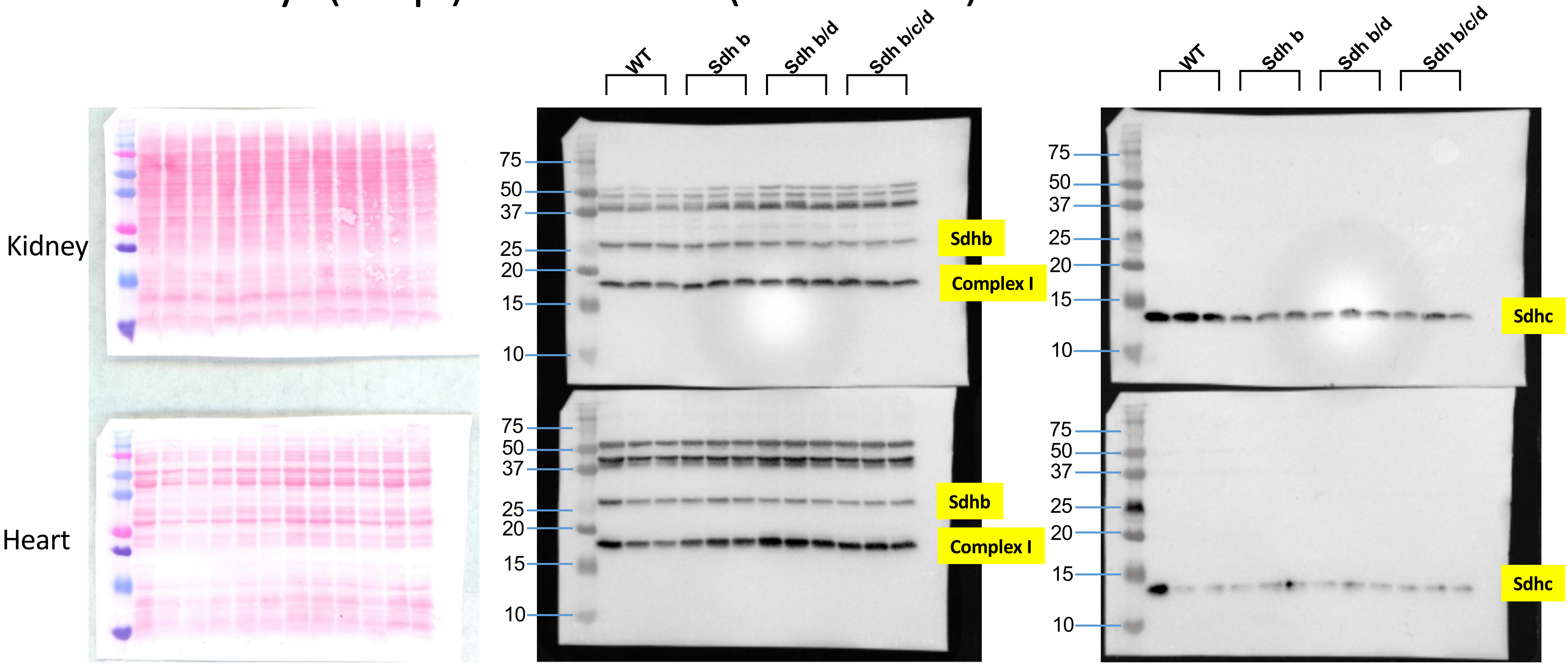

# Full unedited gels for Figure 1
# Brain

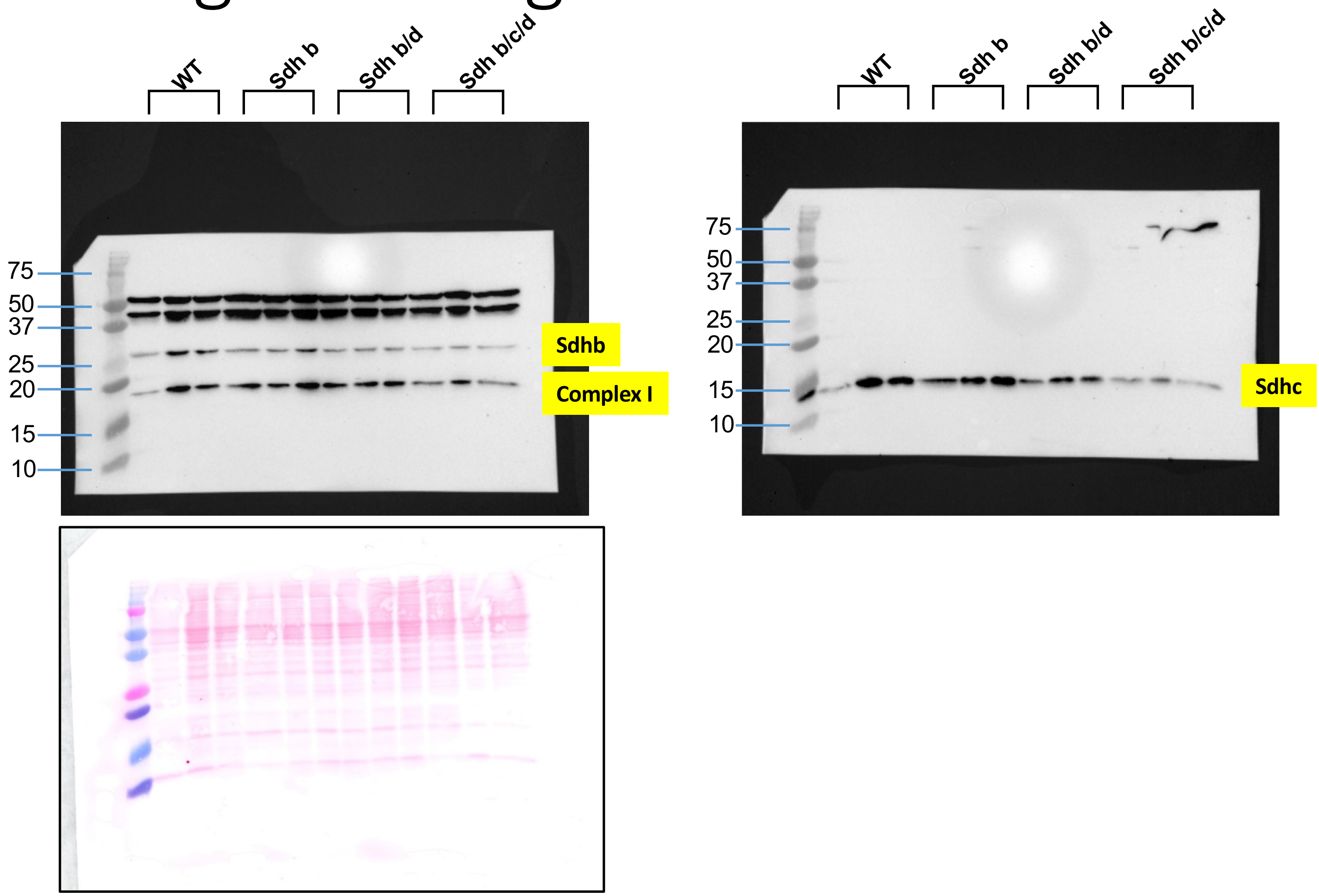


Supplemental Figure 1. Raw western blots for the presentation in Figure 1.

Supplemental Figure 2

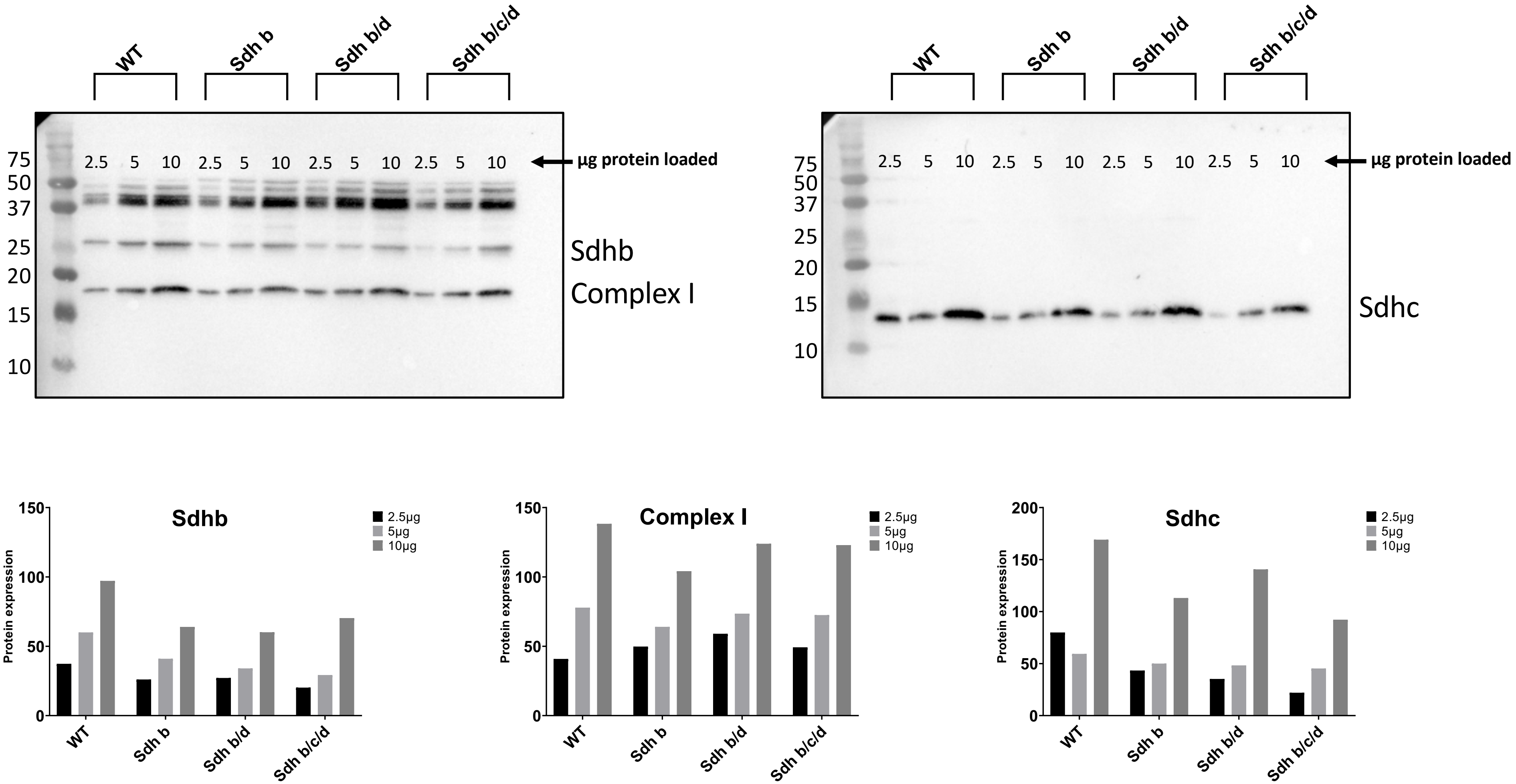


Supplemental Figure 2. Evaluation of linearity in signal-response relationship in western blots.

Supplemental Figure 3

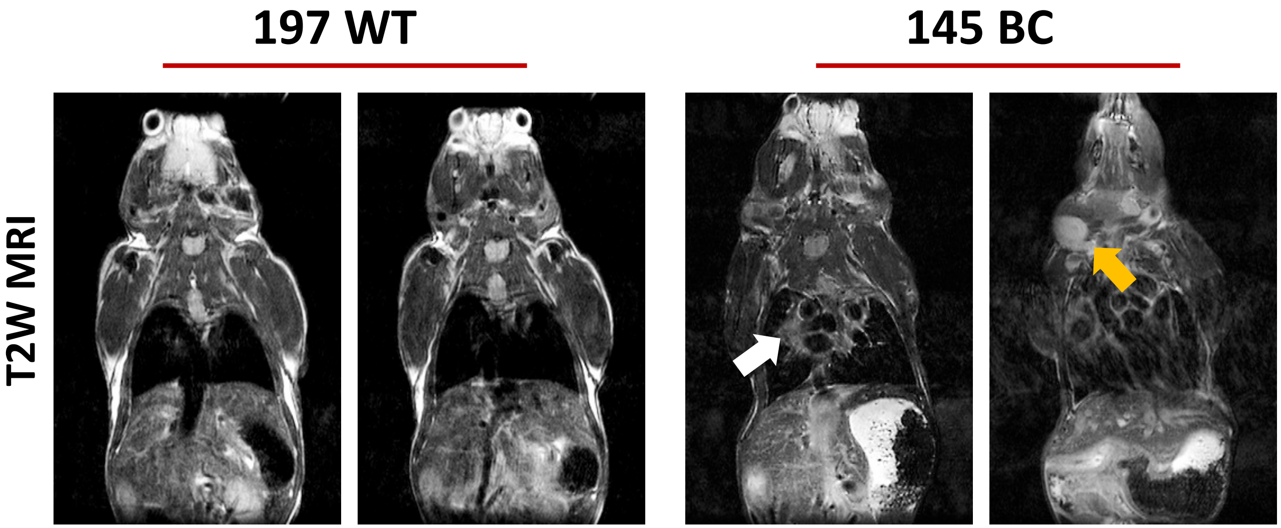


* White arrow points to fibrous changes in lungs suggestive of inflammation.
* Yellow arrow points to enlarged lymph node

Supplemental Figure 3. Coronal T2-weighted MR images of WT (197 WT) andb/c chKO (145b/c) mice (two slices from each animal area shown). Although exposure to chronic hypoxia resulted in non-specific changes in the lungs and lymph nodes, no evidence of tumor development was seen in either group on MR examination.

Supplemental Figure 4

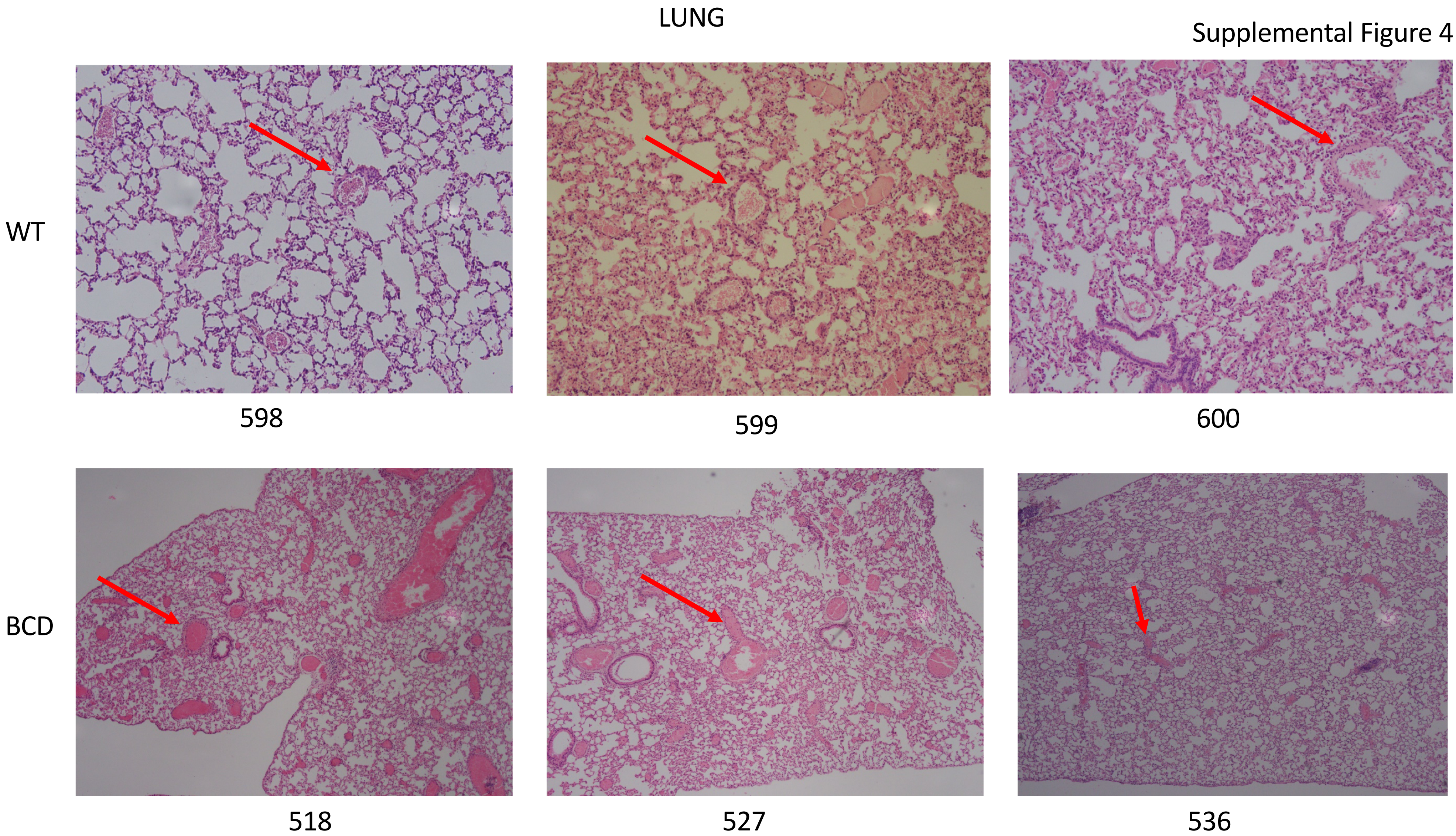

Supplemental Figure 4

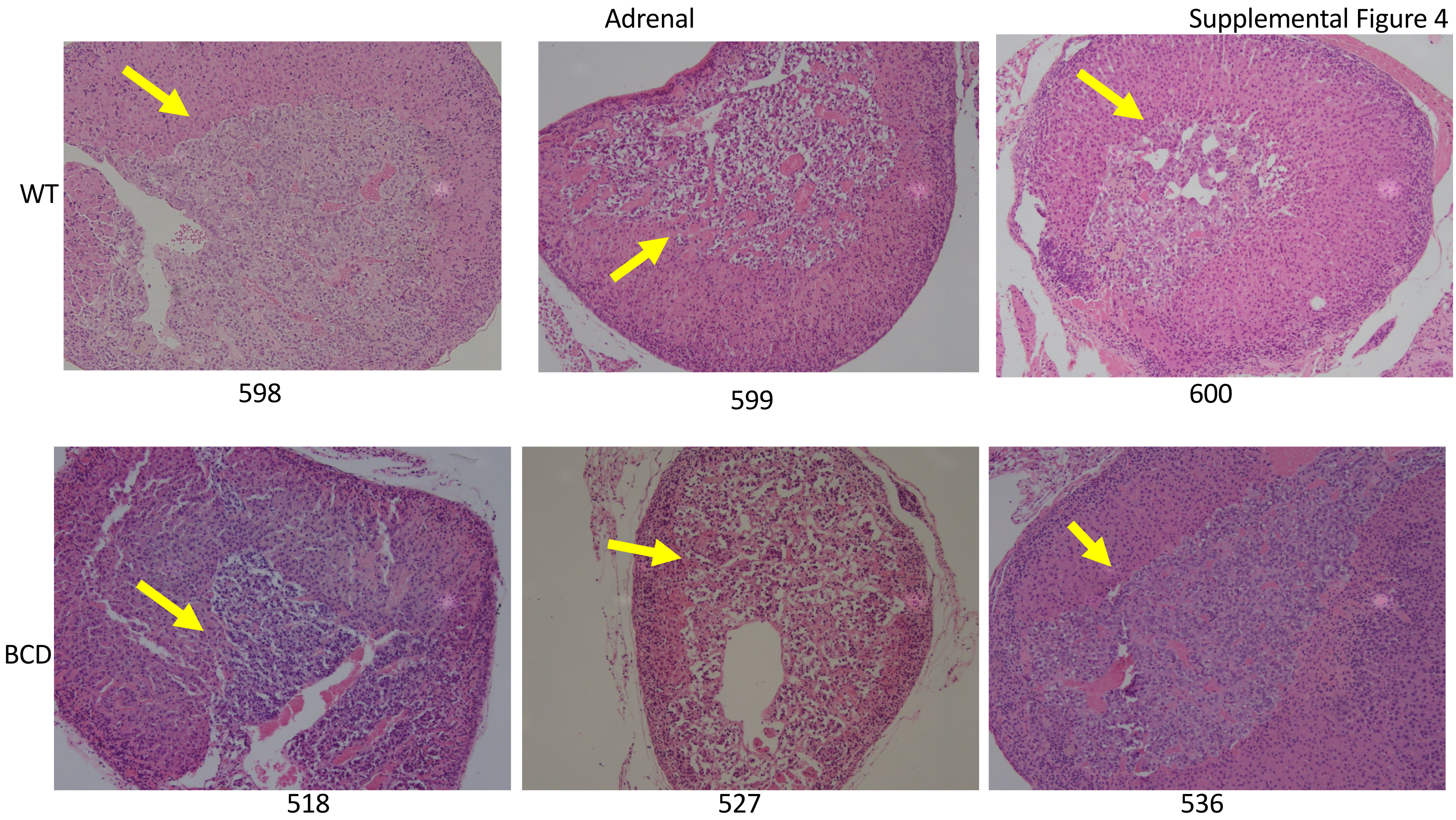


Supplemental Figure 4. Microscopic examination of lungs (A) and adrenal glands (B) show no morphologic evidence of vascular abnormalities or pheochromocytoma. Arrows show lung vessels without morphologic evidence of intimal hyperplasia in lung (A) or point to adrenal medulla (B), surrounded by adrenal cortex in adrenal gland. Images were obtained using Olympus BX45 microscope with 4X or 10X magnification.

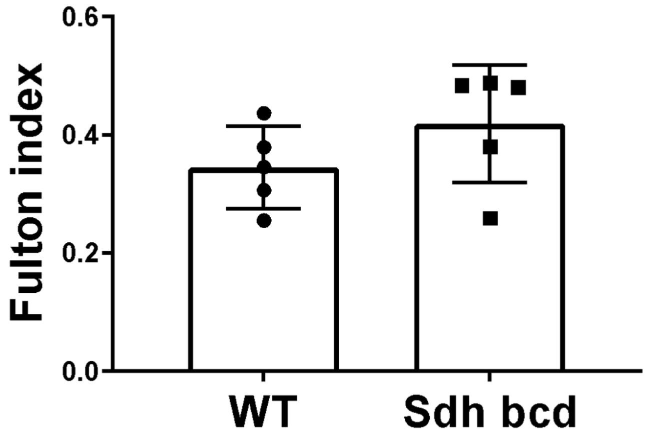


Supplemental Figure 5. Fulton index (ratio of weight of right ventricle to the sum of left ventricle and septum) in group 2 mice showed no statistically significant difference.

Supplemental Figure 6

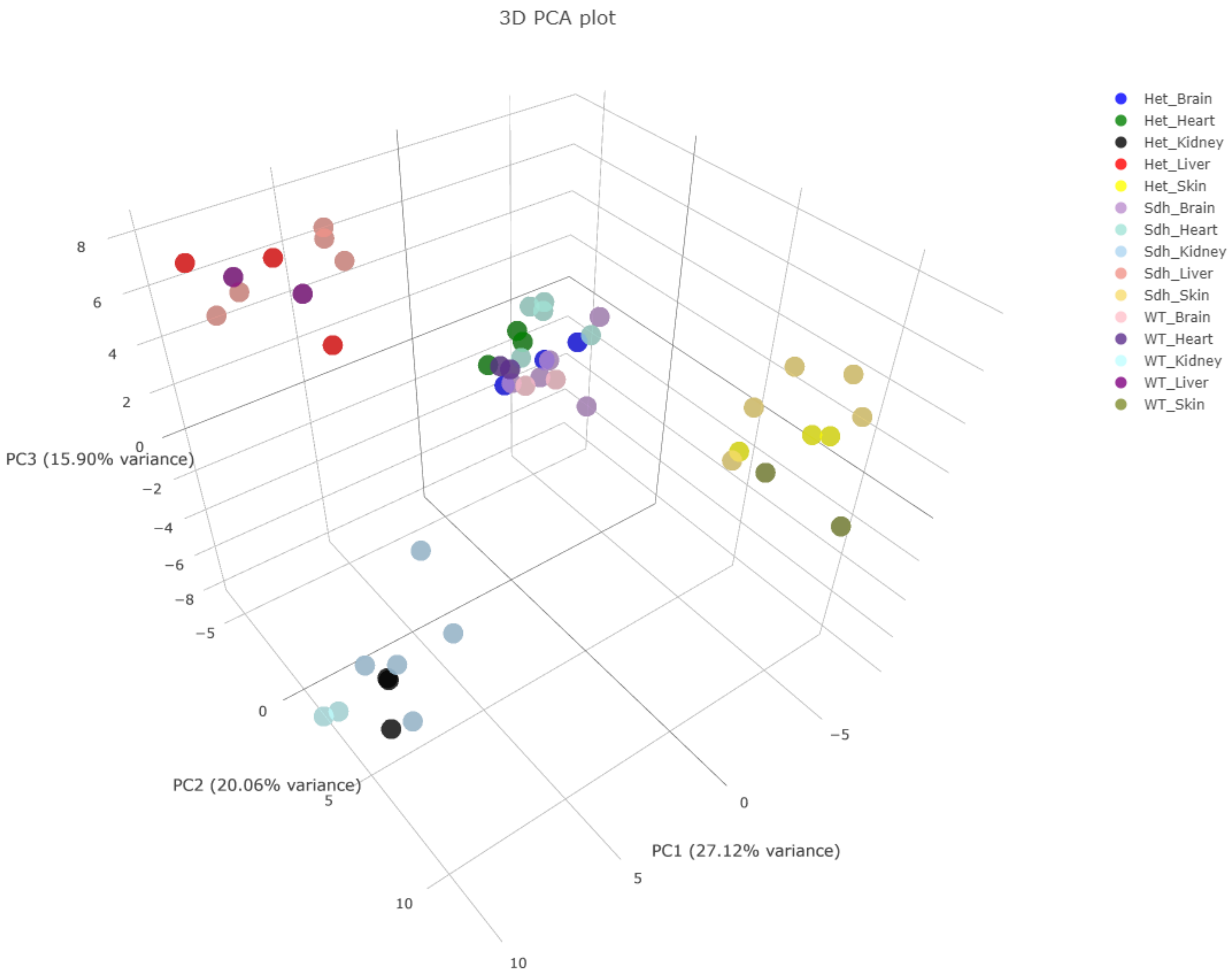


Supplemental Figure 6. Principle component analysis (PCA) shows distinct clustering amongst each tissue type, with the heart samples and the brain samples grouping closely together. Sdh hKO did not cause drastic alterations in tissue specific patterns. WT=Wild-type control, Het=Sdhd single gene hKO, Sdh=Sdhb/c/d triple gene hKO.
